# Hydrogen Sulfide Sustained Release Donor Alleviates Spinal Cord Ischemia–Reperfusion‐Induced Neuron Death by Inhibiting Ferritinophagy‐Mediated Ferroptosis

**DOI:** 10.1111/cns.70366

**Published:** 2025-04-01

**Authors:** Lei Xie, Qiuping He, Hang Wu, Weipeng Shi, Xiao Xiao, Tengbo Yu

**Affiliations:** ^1^ Department of Orthopedic Surgery, Qingdao Municipal Hospital Qingdao University Qingdao China; ^2^ Institute of Sports Medicine and Health Qingdao University Qingdao China; ^3^ Department of Orthopedics, the Affiliated Hospital of Qingdao University Qingdao University Qingdao China; ^4^ Central Laboratories, Qingdao Municipal Hospital University of Health and Rehabilitation Sciences Qingdao China; ^5^ Department of Orthopedic Surgery, Qingdao Municipal Hospital University of Health and Rehabilitation Sciences Qingdao China

**Keywords:** autophagy, ferritinophagy, ferroptosis, hydrogen sulfide, spinal cord ischemia–reperfusion injury

## Abstract

**Aims:**

Spinal cord ischemia–reperfusion injury (SCIRI) is a disastrous complication that cannot be completely prevented in thoracoabdominal aneurysm surgery, leading to sensory and motor dysfunction and even paraparesis, causing tremendous socioeconomic burden. Ferritinophagy is a form of autophagic ferroptosis, which is a contributor to SCIRI. Hydrogen sulfide (H_2_S) has been reported to be neuroprotective in various diseases. However, it remains unclear whether H_2_S alleviates SCIRI‐induced neural death via regulating ferritinophagy‐mediated ferroptosis. The aim of this study was to explore their relationship and interaction in SCIRI.

**Results:**

The results demonstrate that Nissl bodies and motor function were obviously lost in SCIRI rats. Meanwhile, SCIRI led to a significant increase in DHE‐positive neurons, TUNEL‐positive neurons, LC3‐positive neurons, and ferritin‐positive neurons, downregulation of GPx4, Slc7a11, p62, and ferritin expression, and upregulation of LC3 II/I and NCOA4 expression. Additionally, there was upregulation of the level of MDA, GSH, and Fe^2+^. Finally, we found that H_2_S could significantly relieve neuronal death and loss of motor function in SCIRI rats by inhibiting ferritinophagy and ferroptosis.

**Conclusion:**

Ferroptosis and ferritinophagy play a crucial role in the etiopathogenesis of SCIRI, and H_2_S exerts neuroprotection by inhibiting ferritinophagy‐mediated ferroptosis.

## Graphical abstract

H_2_S promotes neuron survival and motor functional recovery via inhibiting neuronal ferritinophagy‐mediated ferroptosis induced by spinal cord ischemic‐reperfusion injury.

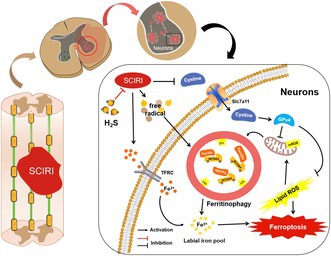

## 1 Introduction

Due to ischemia episodes during thoracoabdominal aneurysm surgery and endovascular aortic repair surgery, spinal cord ischemia–reperfusion injury (SCIRI) remains a complication that cannot be avoided completely, despite advances in surgical technology and skill [1, 2, 3]. It could cause severe dysfunction of sensory and motor functions, and even paraparesis [3]. Intraspinal tumors, spinal trauma, and degeneration can also cause SCIRI, which may result in chronic long‐term or permanent damage if not treated in time [4, 5]. This imposes a tremendous financial burden on patients and society [6]. Hence, it remains warranted to understand in depth the molecular and cellular mechanisms of SCIRI that could identify suitable, effective, and doable treatments.

Ferroptosis is a unique form of programmed cell death (PCD) that performs a crucial function in the elimination of unnecessary and damaged cells [7, 8]. It is different from all main PCDs discovered to date in terms of genetic, biochemical, and morphological characteristics [7]. Ferroptosis is driven by iron‐dependent phospholipid peroxidation, which is characterized by depletion of glutathione (GSH), deactivation of glutathione peroxidase 4 (GPx4), and eventually accumulation of iron‐dependent lipid peroxide, which destroys cellular membrane structures [7, 9]. This essentially is intracellular iron‐induced accumulation of reactive oxygen species and peroxidation of lipids, leading to oxidative damage. There is currently soaring evidence confirming the involvement of ferroptosis in various pathological events, like tumors [10], traumatic and degenerative diseases of the central nervous system [11, 12], osteoporosis and renal allograft ischemia–reperfusion injury [13, 14]. Besides, inhibiting ferroptosis has been shown to effectively prevent ischemia–reperfusion‐induced injury of tissues in diverse experimental models [7].

There is currently growing evidence corroborating that ferritinophagy, a selective cargo autophagic machinery, contributes to the triggering of ferroptosis via ferritin degradation, which leads to cytoplasmic ferrous iron overload, lipid peroxidation, membrane impairment, and cell death [15, 16, 17]. It is essential for regulating intracellular iron content and is characterized by Nuclear Receptor Coactivator 4 (NCOA4), a cargo‐specific receptor that binds to ferritin to form autophagosome and ships it to lysosomes for degradation [13, 17]. Ferritinophagy, causing ferroptosis onset and progression, is at the crossroads of ferroptosis and autophagy, and it might be a critical factor in SCIRI‐induced neuron death. However, the mechanisms and patterns of ferritinophagy in SCIRI have not been thoroughly illuminated and warrant deeper research.

Hydrogen sulfide (H_2_S) was once regarded as a hazardous gas. However, with increasing research, it has been recognized as a crucial endogenous gasotransmitter that exerts a broad physiological function in the body [18, 19]. Recently, the studies on the biology of H_2_S in cells, tissues, and organs have become a popular area, and its importance has also been recognized. It is closely related to the pathological processes of the endocrine [20], cardiovascular [21], gastrointestinal, and nervous systems [22, 23]. Moreover, exogenous H_2_S administration could mitigate SCIRI‐induced neuron death by inhibiting autophagy and reducing oxidative stress, as we previously reported [24]. The connections between ferritinophagy, ferroptosis, and hydrogen sulfide have not been reported. Moreover, it is crucial for shedding light on the pathogenesis of SCIRI and paving the way for H_2_S‐based innovative therapeutic interventions [25]. Therefore, we attempted to explore whether the therapeutic effect of H_2_S is related to ferritinophagy‐mediated ferroptosis.

Here, we found that SCIRI could induce autophagic ferritin degradation and eventually cause iron‐dependent ferroptosis. Moreover, the results of our findings indicate that H_2_S could attenuate neuron death by inhibiting ferritinophagy and ferroptosis. Shortly, our study demonstrates that ferritinophagy‐mediated ferroptosis contributes to the etiopathogenesis of SCIRI, and H_2_S may be a potential and feasible treatment for SCIRI.

## 2 Methods

### 2.1 Animals

All male Sprague–Dawley (SD) rats (188–200 g weight) in this study were obtained from the Animal Laboratory of Beijing Charles River Corporation (Beijing, China). The rats were housed with water and commercial diet available ad libitum in an air‐conditioned room with constant temperature and humidity and a 12‐h light/dark cycle and were acclimated to their surroundings for 1 week prior to experiments. All animal operations and care procedures were approved by the Qingdao University Laboratory Animal Welfare Ethics Committee (No. 20230820SD20231212042).

### 2.2 Spinal Cord Ischemia–Reperfusion Injury (SCIRI) Model

All rats were neurologically intact before the experiment. The SCIRI model was built as we previously described [26]. In brief, the rats were anesthetized and fixed supine, and a midline abdominal incision was used. The abdominal aorta was clamped under the right renal artery near the heart using a 50 g aneurysm clip for 60 min (which was not clamped in sham‐operated rats). After the operation, the rat was placed in a box at 28°C to recover from anesthesia and subsequently placed in a separate cage with food and water available at will.

### 2.3 Drugs Preparation and Procedures

3‐Methyladenine (3‐MA; 189,490) and ferrostatin‐1 (SML0583) were purchased from Sigma‐Aldrich. GYY4137 (HY‐107632), rapamycin (AY‐22989), and erastin (HY‐15763) were purchased from MedChemExpress. GYY4137, 3‐MA, erastin, ferrostatin‐1 (Fer‐1), and rapamycin (Rapa) were dissolved in dimethyl sulfoxide to yield a stock solution and further diluted in phosphate‐buffered saline (PBS) for the final dose before intraperitoneal injection.

The Sham group underwent the surgical procedure without aortic clipping. The SCIRI group was modeled with SCIRI, and an equivalent amount of vehicle solution was injected intraperitoneally immediately after reperfusion. The rats in the SCIRI+Fer‐1 group and SCIRI+3‐MA group also received the same procedure as the SCIRI group, but were treated with Fer‐1 (2 mg/kg) [13] or 3‐MA (2.5 mg/kg) [24] immediately after reperfusion, respectively. The rats in the SCIRI+GYY4137 group underwent the same surgical procedure as the SCIRI group, but were treated with GYY4137 (50 mg/kg) 30 min before the onset of spinal cord reperfusion [27]. The rats in the SCIRI + GYY4137 + Rapa group and SCIRI + GYY4137 + Erastin group also received the same procedure as the SCIRI + GYY4137 group, but were treated with rapamycin (0.5 mg/kg) [24] or erastin (20 mg/kg) [28], 30 min before GYY4137 administration, respectively.

### 2.4 Neurological Function Analysis

The Basso, Beattie, and Bresnahan (BBB) open‐field locomotor scale ranges from 0 (complete paralysis) to 21 (normal locomotion) and was used to measure locomotor recovery after SCIRI [29]. The BBB scores at 1, 6, 12, and 24 h after reperfusion were recorded by two experienced investigators who were blinded to the experimental design.

### 2.5 Malondialdehyde, Glutathione, and Iron Assay

According to the manufacturer's instructions, the concentration of the lipid peroxidation products malondialdehyde (MDA), glutathione (GSH), and iron was measured in the spinal cord lysates using the corresponding kits, respectively. MDA content assay kit (bc0025), GSH content assay kit (bc1175), and tissue iron content assay kit (bc4355) were purchased from Solarbio.

### 2.6 Antibodies

The antibodies used for immunofluorescence and immunohistochemical are as follows: anti‐NeuN (1:400, 94,403) and anti‐LC3B (1:200, 83,506) were purchased from Cell Signaling Technology. anti‐NeuN (1:200, 26975‐1‐AP) and anti‐GPx4 (1:200, 30388‐1‐AP) were purchased from Proteintech. anti‐ferritin (1:100, ab75973) and anti‐TFRC (1:1000, ab269513) were purchased from Abcam. Alexa Fluor 488‐labeled goat anti‐rabbit IgG (H + L) (1:300, A0423), Alexa Fluor 488‐labeled goat anti‐mouse IgG (H + L) (1:300, A0428), Alexa Fluor 555‐labeled donkey anti‐rabbit IgG (H + L) (1:300, A0453), and Alexa Fluor 555‐labeled donkey anti‐mouse IgG (H + L) (1:300, A0460) were purchased from Beyotime. The antibodies used for Western blotting are as follows: anti‐GPx4 (1:500, A11243) and anti‐SLC7A11/xCT (1:500, A13685) were purchased from ABclonal. anti‐ferritin (1:1000, ab75973), anti‐NCOA4 (1:1000, ab314553), anti‐SQSTM1/p62 (1:10000, ab109012), and anti‐LC3B (1:1000, ab192890) were purchased from Abcam. anti‐β‐actin (1:5000, BM0627), HRP Conjugated AffiniPure goat anti‐mouse IgG (H + L) (1:5000, BA1050), and HRP Conjugated AffiniPure goat anti‐rabbit IgG (H + L) (1:5000, BA1054) were purchased from Boster.

### 2.7 Immunofluorescence Staining

The protocol for immunofluorescence staining was based on our previously described method [24]. In brief, the spinal cord frozen sections were washed with PBS, permeabilized with 1% Triton X‐100, blocked with 5% bovine serum albumin (BSA), incubated with primary antibody, and then incubated with the corresponding secondary antibodies conjugated with Alexa Fluor‐labeled. Finally, the sections were mounted with ProLong Gold antifade reagent with DAPI to label the nuclei (Invitrogen, P36935). Traced sections were examined with a Zeiss microscope (Axioscope 5). Images and their colocalization or fluorescence intensity were quantified using ZEN Lite Viewer and ImageJ software.

### 2.8 Immunohistochemical Staining

The staining protocol was based on our previous report [30]. Briefly, paraffin sections were deparaffinized, rehydrated, and underwent antigen retrieval. Then the sections were incubated in hydrogen peroxide, blocked, incubated with primary antibody, and then incubated with the corresponding SABC‐HRP kit (Beyotime, P0612/P0615). Positive staining was visualized with DAB (Beyotime, P0202) and counterstained with hematoxylin. Traced sections were examined with a Nikon microscope (Ni‐U). Images and mean integrated optical density were quantified using NIS‐Elements Viewer and ImageJ.

### 2.9 Nissl Body Staining

The staining protocol was based on the instructions of the manufacturer of Nissl staining solution (Beyotime, C0117). In brief, paraffin sections were deparaffinized, rehydrated, and incubated in Nissl staining solution. Then, sections were dehydrated, transparent, and mounted. Traced sections were examined with a Nikon microscope (Ni‐U). Images and amounts were quantified using NIS‐Elements Viewer and ImageJ.

### 2.10 Dihydroethidium Staining

Dihydroethidium (DHE) staining was used to assess the level of oxidative stress as previously reported [31]. Briefly, the frozen spinal cord sections were incubated with the fluorescent dye DHE (Beyotime, S0063). Traced sections were examined with a Zeiss microscope. Images and fluorescence intensity were quantified using ZEN Lite Viewer and ImageJ software.

### 2.11 Terminal Deoxynucleotidyl Transferase‐Mediated dUTP Nick‐End Labeling (TUNEL) Staining

According to the manufacturer's instructions of the TUNEL kit (Roche, 12156792910) to detect apoptotic cells. In brief, the frozen sections were washed in PBS, permeabilized, blocked, and incubated with the TUNEL reaction mixture. Traced sections were examined with a Zeiss microscope. Immunofluorescence images and their colocalization were quantified using ZEN Lite Viewer and ImageJ software.

### 2.12 Western Blotting

The protocol was based on previously described [27]. Briefly, the spinal cords were treated with ice‐cold RIPA (Beyotime, P0013C) containing a protease and phosphatase inhibitor cocktail (Beyotime, P1050), and the concentration of protein was measured with the BCA assay kit (Beyotime, P0012). Aliquots of protein were separated by sodium dodecyl sulfate–polyacrylamide gel electrophoresis and transferred to a polyvinylidene difluoride filter membrane (0.2 μm, Millipore, ISEQ00010). Subsequently, the membranes were blocked, incubated with the primary antibody, and incubated with the corresponding secondary antibody. Finally, the chemiluminescence results were recorded using an imaging system (FUSION SOLO S, VILBER). The bands relative intensities were calculated by ImageJ software.

### 2.13 Statistical Analysis

All data were statistically analyzed using GraphPad Prism 9. The data are presented as the mean ± standard deviation (mean ± SD) of at least three independent experiments in this study. The normality of the data distribution was evaluated using the Shapiro–Wilk test. If the data were normally distributed, Student's *t*‐test or one‐way analysis of variance (ANOVA) was used to assess group differences. For data that were not normally distributed, a non‐parametric test (Kruskal‐Wallis test) was applied. Two‐way ANOVA and Tukey's post hoc test were employed to evaluate differences among multiple groups with repeated measures. For all statistical tests, a *p*‐value < 0.05 was considered statistically significant.

## 3 Results

### 3.1 SCIRI‐Induced Cell Death, Neuron Loss, and Locomotor Function Impairment

**FIGURE 1:**
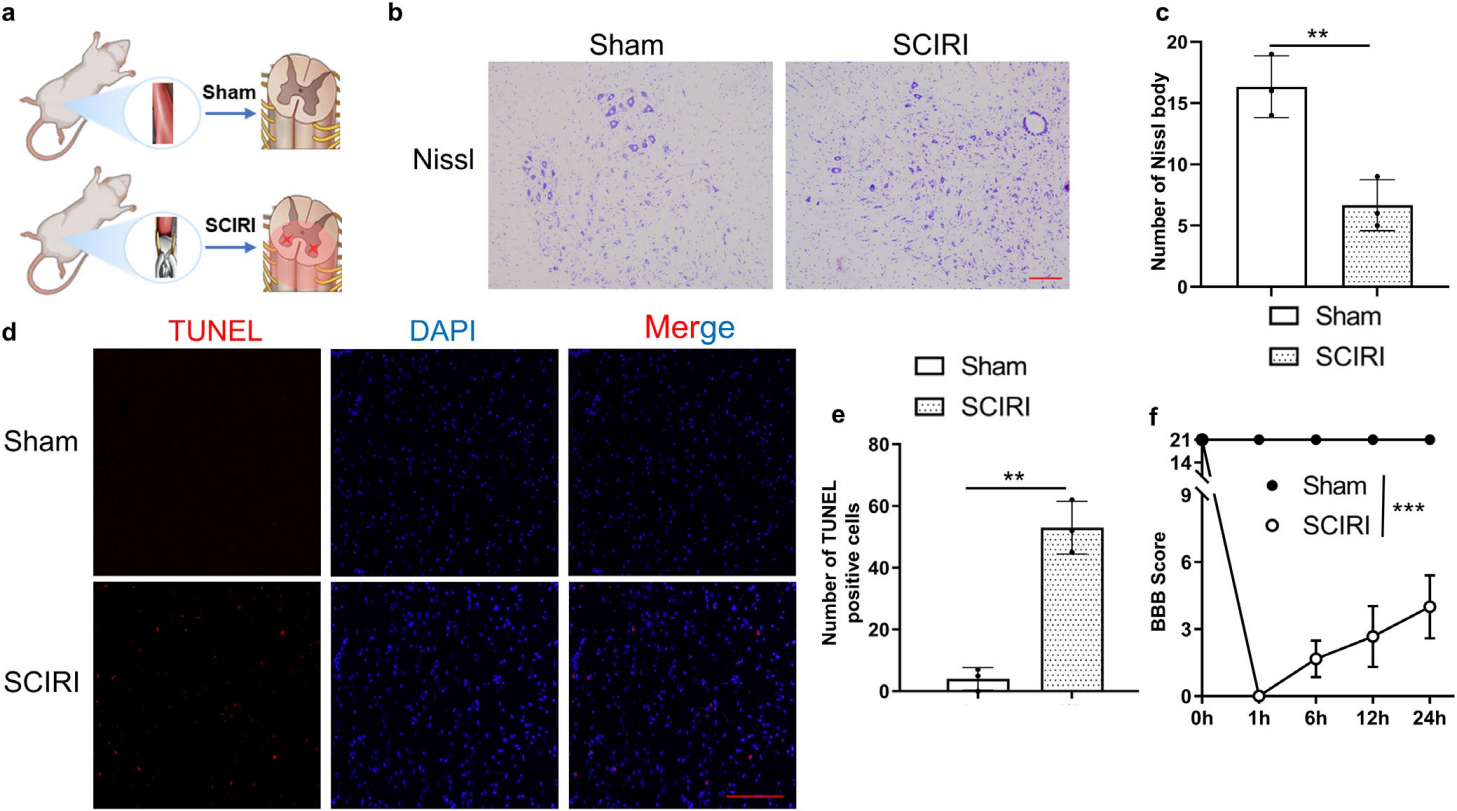
Ischemia–reperfusion induced cell death in SCIRI rats. (a) Schematic diagram depicts the operation for SCIRI rats. (b) The Nissl staining of the anterior horn of the spinal cord in the Sham group and SCIRI group (100×, scale bar = 200 μm). (c) Statistical analyses of Nissl bodies (*n* = 3 rats per group). (d) The TUNEL staining of the spinal cord in each group (200×, scale bar = 100 μm). (e) Statistical analyses of the count of TUNEL‐positive cells in the spinal cord (*n* = 3 rats per group). (f) The BBB scores for different groups at each time point (*n* = 6 rats per group). **p < 0.01; ***p < 0.001.

To verify the existence of neuron damage in a rat model of SCIRI (Figure 1a), we performed Nissl staining to evaluate changes in neuronal viability, a TUNEL assay to evaluate the viability of cells around the anterior horn, and the motor function was assessed by the BBB score.

The results of Nissl staining revealed that the number of Nissl bodies in the anterior horn of the spinal cord was significantly decreased in the SCIRI group compared to the Sham group (Figure 1b,c). In addition, the numbers of TUNEL‐positive cells were obviously increased in the SCIRI group compared to the Sham group (Figure 1d,e). Moreover, BBB scores demonstrated that motor function was largely decreased after reperfusion (Figure 1f). In summary, we generated a SCIRI rat model that was characterized by neurological dysfunction, which was consistent with increased cell death and loss of motor neurons.

### 3.2 Ferroptosis Was Activated by SCIRI and Promoted Neuron Death

**FIGURE 2:**
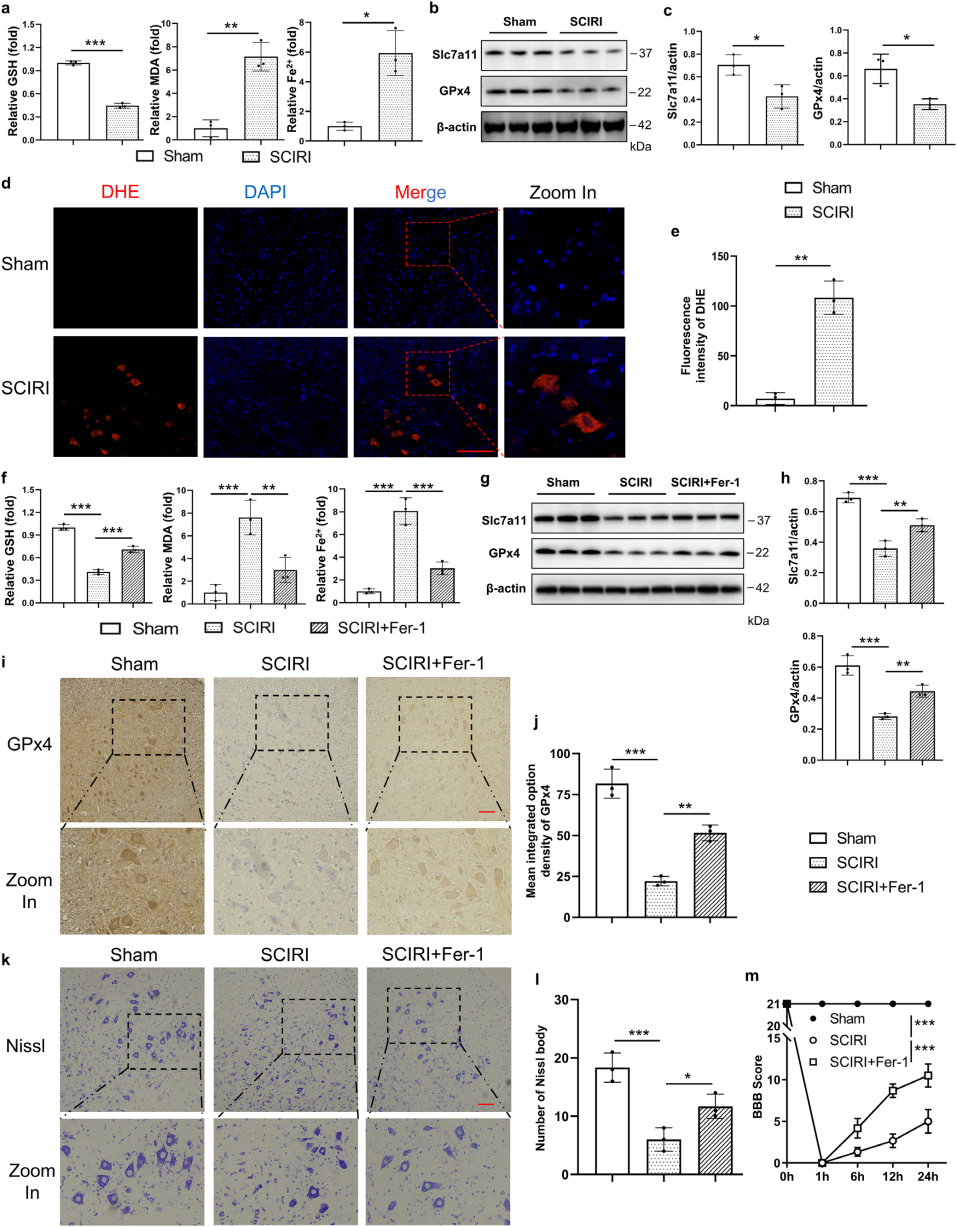
Ferroptosis was activated by SCIRI and promoted neuron death. (a) Relative concentration of GSH, MDA, and Fe^2+^ of the spinal cord in the Sham group and SCIRI group (*n* = 6 rats per group). (b) Western blotting was used to detect the expression of the protein of Slc7a11 and GPx4 of the spinal cord in each group. (c) Densitometric analysis and quantification of Slc7a11/Actin and GPx4/Actin (*n* = 6 rats per group). (d) The DHE staining of the spinal cord in each group (200×, scale bar = 100 μm). (e) Quantitative fluorescence intensity of DHE (*n* = 3 rats per group). (f) Relative concentrations of GSH, MDA, and Fe^2+^ of the spinal cord in the Sham group, SCIRI group, and SCIRI + Fer‐1 group (*n* = 6 rats per group). (g) Western blotting was used to detect the expression of the protein of Slc7a11 and GPx4 of the spinal cord in each group. (h) Densitometric analysis and quantification of Slc7a11/Actin and GPx4/Actin (*n* = 6 rats per group). (i) The GPx4 immunohistochemistry staining of the spinal cord in each group (200×, scale bar = 100 μm). (j) Statistical analysis of the mean integrated option density of GPx4 (*n* = 3 rats per group). (k) The Nissl staining of the anterior horn of the spinal cord in each group (200×, scale bar = 100 μm). (l) Statistical analyses of Nissl bodies (*n* = 3 rats per group). (m) The BBB scores of different groups at each time point (*n* = 6 rats per group). *p < 0.05; **p < 0.01; ***p < 0.001.

Substantial evidence revealed the crucial role of ferroptotic machinery in driving cell death [32, 33]. Our previous study showed that SCIRI could induce lipid peroxidation, which is one of the key events of ferroptosis. Herein, we assumed that ferroptosis might contribute to neuron death in SCIRI. To verify this assumption, we assessed the concentrations of GSH, MDA, and Fe^2+^, and these are typical indicators for ferroptosis. As expected, the results demonstrated that the higher concentrations of MDA and Fe^2+^, and the lower GSH in the SCIRI group compared to the Sham group. This means that ferroptosis events, depletion of GSH, peroxidation of lipid, and accumulation of Fe^2+^, were indeed triggered by SCIRI (Figure 2a). The dihydroethidium (DHE) staining results also proved this (Figure 2d,e). Moreover, we conducted Western blotting for the expression of GPx4 and solute carrier family 7 member 11 (Slc7a11), both considered critical for anti‐ferroptosis (Figure 2b). The results showed that the expression of Slc7a11 and GPx4 was obviously decreased in the SCIRI group (Figure 2c). Together, these findings suggested that ferroptosis was activated by SCIRI.

To further clarify whether ferroptosis was required for neuron death, we inhibited ferroptosis by Ferrostatin‐1 (Fer‐1, a lipid ROS scavenger), which is an inhibitor of ferroptosis. The results showed that ferroptosis events were reversed by Fer‐1 (Figure 2f). Moreover, the expression of Slc7a11 and GPx4 was obviously increased in SCIRI rats with Fer‐1 treatment compared to the SCIRI group (Figure 2g,h). The GPx4 immunohistochemistry staining results also proved this (Figure 2i,j). Besides, the number of Nissl bodies and BBB scores were significantly increased with Fer‐1 treatment compared to the SCIRI group (Figure 2k–m), and these were consistent with the results of TUNEL staining (Figure S1). Taken together, our findings suggested that SCIRI initiated ferroptosis, which might be crucial for inducing neuronal death.

### 3.3 Autophagy Was Activated by SCIRI and Promoted Neuronal Death

**FIGURE 3:**
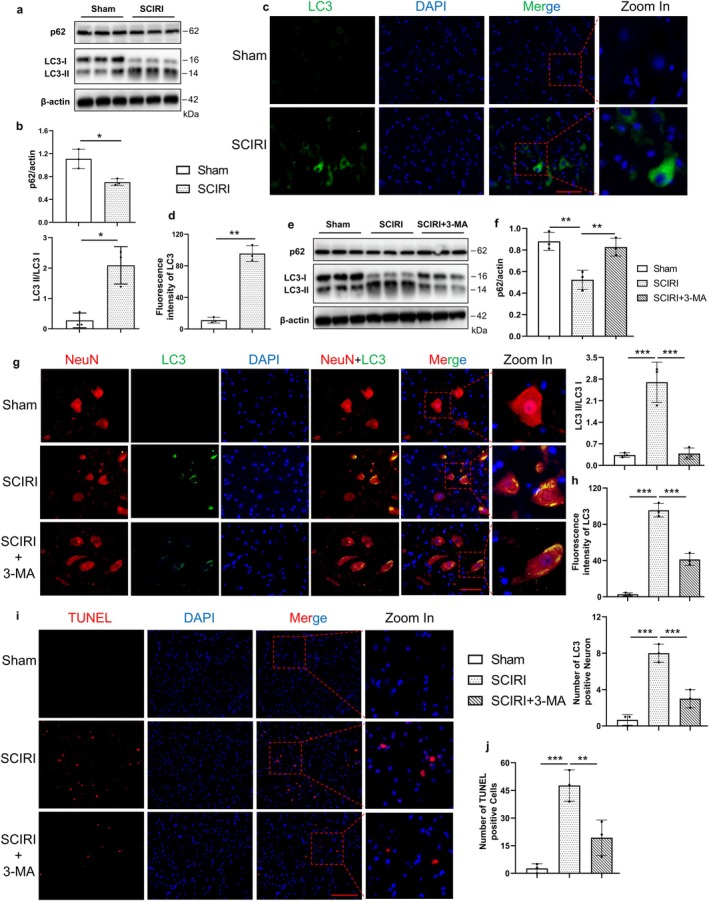
Autophagy was activated by SCIRI and promoted neuronal death. (a) Western blotting was used to detect the protein expression of p62 and LC3 of the spinal cord in the Sham group and SCIRI group. (b) Densitometric analysis and quantification of p62/Actin and LC3II/I (*n* = 6 rats per group). (c) The LC3 immunofluorescence staining of the spinal cord in each group (400×, scale bar = 50 μm). (d) Quantitative fluorescence intensity of LC3 (*n* = 3 rats per group). (e) Western blotting was used to detect the protein expression of p62 and LC3 of the spinal cord in the Sham group, SCIRI group, and SCIRI + 3‐MA group. (f) Densitometric analysis and quantification of p62/Actin and LC3II/I (*n* = 6 rats per group). (g) The NeuN and LC3 immunofluorescence colocalization staining of the spinal cord in each group, red (NeuN), green (LC3), and blue (DAPI) (400×, scale bar = 50 μm). (h) Quantitative fluorescence intensity of LC3 and quantification of the number of LC3‐positive neurons (*n* = 3 rats per group). (i) The TUNEL staining of the spinal cord in each group (200×, scale bar = 100 μm). (j) Statistical analyses of the count of TUNEL‐positive cells in the spinal cord (*n* = 3 rats per group). *p < 0.05; **p < 0.01; ***p < 0.001.

Our previous study reported that SCIRI could activate autophagy, which has been identified to play a key role in driving cells toward ferroptotic death [30, 34, 35]. We conducted Western blotting for the expression of LC3 and p62, which are two typical markers of autophagy (Figure 3a). The results showed that the SCIRI group decreased the expression of p62 and upregulated the protein ratio of LC3‐II/I compared to the sham group (Figure 3b), and these were consistent with the results of immunofluorescence staining of LC3 (Figure 3c,d). Together, these findings suggested that autophagy was activated in SCIRI rats.

To elucidate whether autophagy was necessary for neuron death, the autophagy inhibitor 3‐methyladenine (3‐MA) was further used. Western blotting analysis showed that treatment with 3‐MA reduced the LC3‐II/I ratio and increased the p62 protein level compared to the SCIRI group (Figure 3e,f). We also performed immunofluorescence colocalization of LC3 with NeuN to investigate LC3 expression and localization. The results indicated that autophagy occurred in neurons following SCIRI and was inhibited by 3‐MA treatment (Figure 3g,h). Additionally, the number of TUNEL‐positive cells significantly decreased in the SCIRI rats with 3‐MA treatment (Figure 3i,j). Altogether, these results suggested that SCIRI activated autophagy, which was critical for inducing neuronal death.

### 3.4 Ferritinophagy Was Activated by SCIRI

**FIGURE 4:**
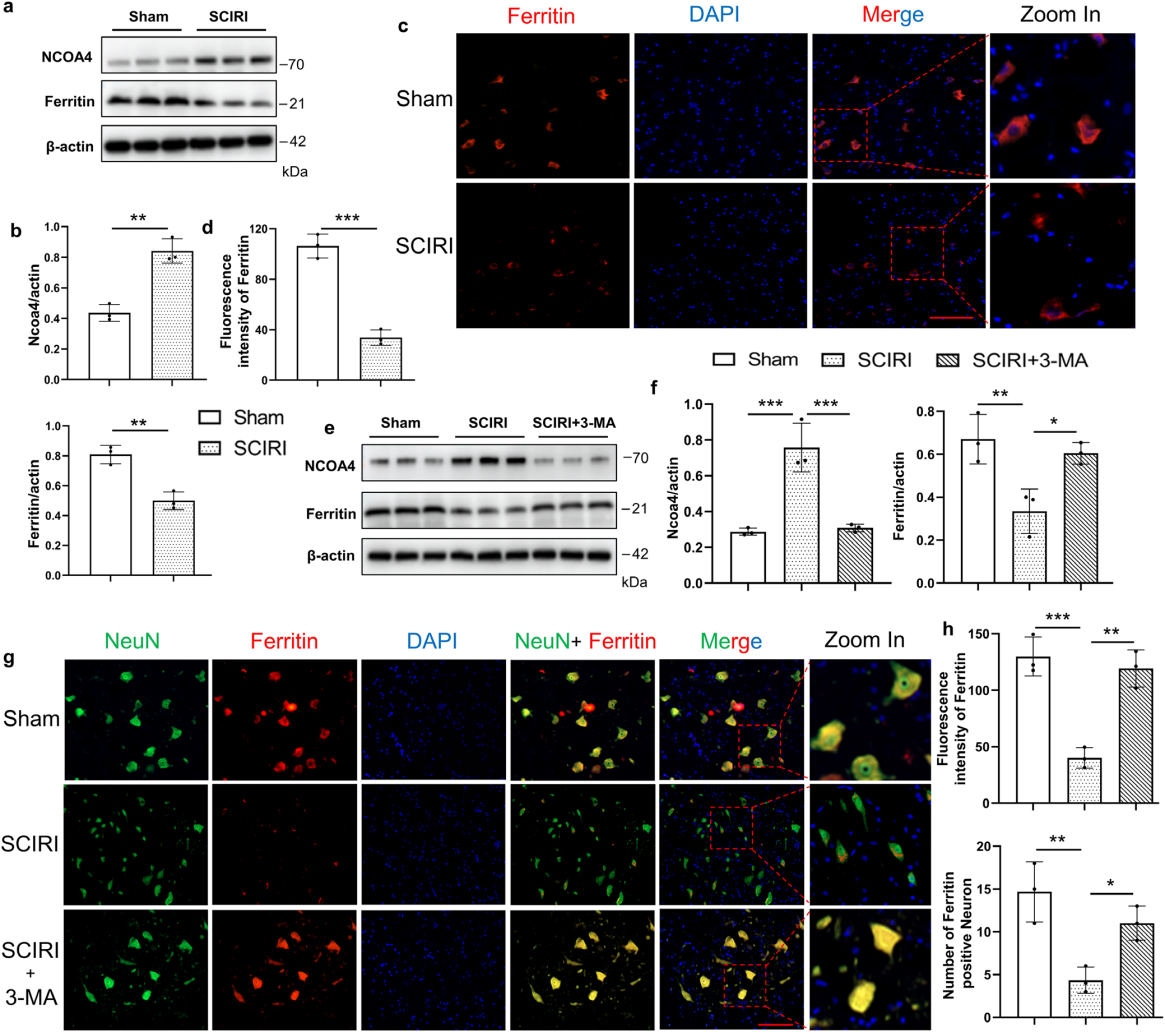
Ferritinophagy was activated by SCIRI. (a) Western blotting was used to detect the protein expression of NCOA4 and ferritin of the spinal cord in the Sham group and SCIRI group. (b) Densitometric analysis and quantification of Ncoa4/Actin and ferritin/Actin (*n* = 6 rats per group). (c) The ferritin immunofluorescence staining of the spinal cord in each group (200×, scale bar = 100 μm). (d) Quantitative fluorescence intensity of ferritin (*n* = 3 rats per group). (e) Western blotting was used to detect the protein expression of NCOA4 and ferritin of the spinal cord in the Sham group, SCIRI group, and SCIRI + 3‐MA group. (f) Densitometric analysis and quantification of Ncoa4/Actin and ferritin/Actin (*n* = 6 rats per group). (g) The NeuN and ferritin immunofluorescence colocalization staining of the spinal cord in each group, red (NeuN), green (ferritin) and blue (DAPI) in each group (200×, scale bar = 100 μm). (h) Quantitative fluorescence intensity of ferritin and quantification of the number of ferritin‐positive neurons (*n* = 3 rats per group). *p < 0.05; **p < 0.01; ***p < 0.001.

It has been demonstrated that NCOA4 selectively autophagically degrades ferritin, the major protein for intracellular iron storage, thereby accumulating labile iron and promoting ferroptosis [13, 36]. So, we focus on NCOA4‐mediated ferritinophagy in SCIRI. we conducted Western blotting for the expression of ferritin and NCOA4 (Figure 4a), which are vital proteins for NCOA4‐mediated ferritinophagy. The results showed that the SCIRI group had decreased the expression of ferritin and had increased the expression of NCOA4 compared to the sham group (Figure 4b). In addition, the ferritin immunofluorescence staining was consistent with the above results (Figure 4c,d).

To further verify that ferritinophagy was involved in SCIRI‐induced cell death, we inhibited autophagy with 3‐MA. Western blotting results showed that 3‐MA treatment increased the expression of ferritin and decreased the expression of NCOA4 compared to the SCIRI group (Figure 4e,f). Furthermore, the immunofluorescence colocalization of NeuN and ferritin supported the above findings (Figure 4g). It showed that with 3‐MA treatment, the ferritin expression was increased in neurons compared to the SCIRI group (Figure 4h). Collectively, these results imply that NCOA4‐mediated autophagic‐dependent ferroptosis might be the pivotal process for SCIRI‐induced neuron death.

### 3.5 Hydrogen Sulfide Attenuated Neuron Death by Inhibiting SCIRI‐Induced Autophagic‐Mediated Ferroptosis

**FIGURE 5:**
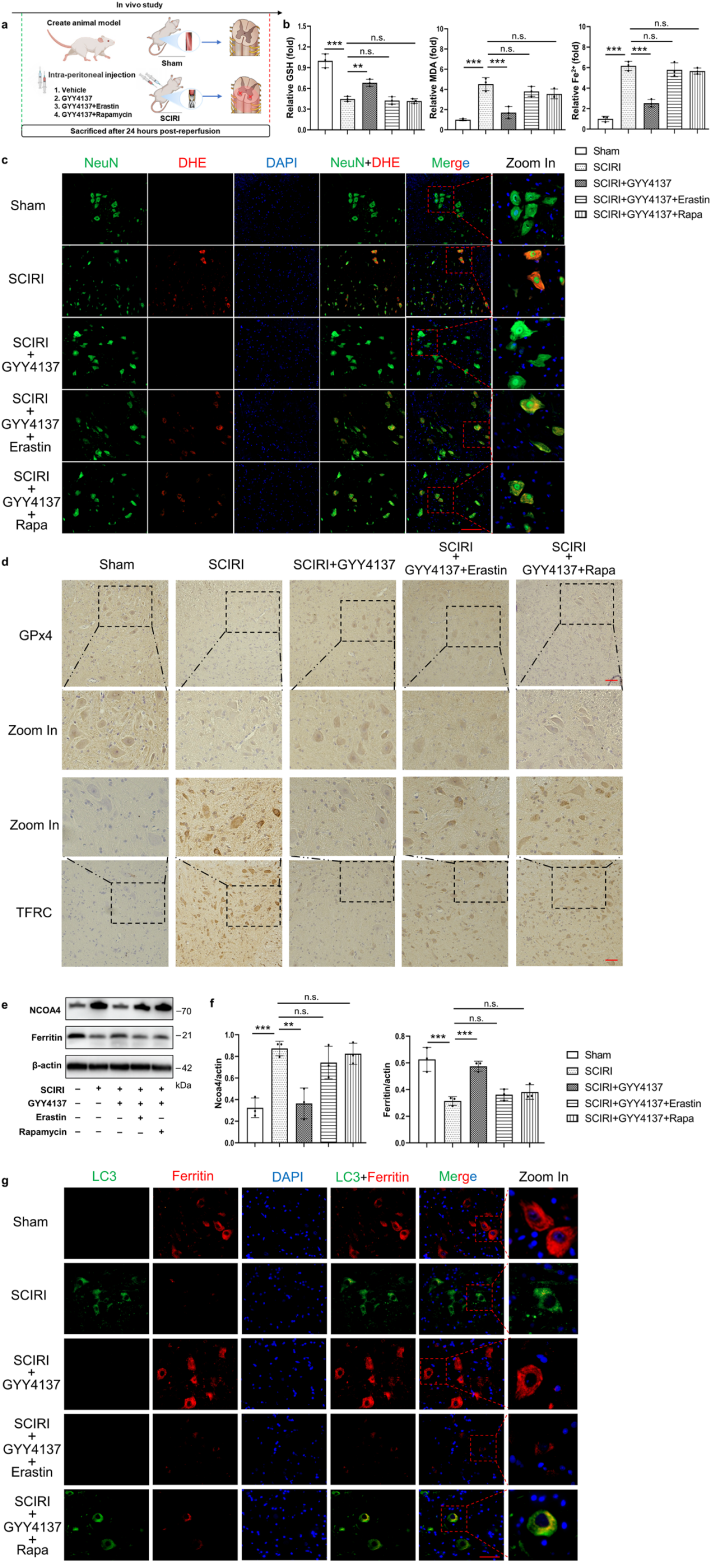
H_2_S could inhibit ferritinophagy and ferroptosis activated by SCIRI. (a) Schematic diagram depicts the operation and different treatments for rats. (b) Relative concentrations of GSH, MDA, and Fe^2+^ of the spinal cord in the Sham, SCIRI, SCIRI + GYY4137, SCIRI + GYY4137 + Erastin, and SCIRI + GYY4137 + Rapa group (*n* = 6 rats per group). (c) The NeuN and DHE immunofluorescence colocalization staining of the spinal cord in each group, red (DHE), green (NeuN) and blue (DAPI) (200×, scale bar = 100 μm, *n* = 3 rats per group). (d) The GPx4 and TFRC immunohistochemistry staining of the spinal cord in each group (200×, scale bar = 100 μm, *n* = 3 rats per group). (e) Western blotting was used to detect the protein expression of NCOA4 and ferritin of the spinal cord in each group. (f) Densitometric analysis and quantification of Ncoa4/Actin and ferritin/Actin (*n* = 6 rats per group). (g) The LC3 and ferritin immunofluorescence colocalization staining of the spinal cord in each group, red (ferritin), green (LC3) and blue (DAPI) (200×, scale bar = 100 μm, *n* = 3 rats per group). **p < 0.01; ***p < 0.01; n.s., not significant.

We previously reported that H_2_S could inhibit autophagy and reduce oxidative stress in SCIRI rats [24], and these are key events of ferritinophagy and ferroptosis [17, 34]. In addition, we found that GYY4137, a sustained release H_2_S donor, not only regulated autophagy but also ferroptosis (Figures S2, S3). Consequently, it is reasonable to hypothesize that the therapeutic effect of H_2_S in SCIRI might be associated with inhibited ferritinophagy and ferroptosis. To test our hypothesis (Figure 5a), we first assessed the concentrations of GSH, MDA, and Fe^2+^. The results showed that depletion of GSH, peroxidation of lipids, and accumulation of Fe^2+^ were reversed with GYY4137 treatment compared to SCIRI, whereas these effects were abolished by the combination of GYY4137 with rapamycin (autophagy activator) or erastin (ferroptosis inducer) (Figure 5b). The DHE staining results also supported these findings (Figure 5c and Figure S4a). Moreover, we performed Western blotting to assess the degree of ferroptosis. Compared to the SCIRI group, GYY4137 treatment significantly reduced the expression of GPx4 and slc7a11, whereas GYY4137 combined with rapamycin or erastin had no such effect (Figure S3). These were consistent with the results of immunohistochemistry staining GPx4 and TFRC (Figure 5d and Figure S4b).

Then, the expression of Ferritin and NCOA4 proteins was reversed in the GYY4137 treatment group compared to the SCIRI group, but these were not different in GYY4137 combined with rapamycin or erastin compared to the SCIRI group. (Figure 5e,f). Additionally, the LC3 and Ferritin immunofluorescence colocalization staining was used to detect ferritinophagy (Figure 5g). As increased LC3 expression occurred, Ferritin expression decreased in the SCIRI group compared to the sham group, and these changes could be reversed with GYY4137 treatment (Figure S4c). Rapamycin or erastin could abolish the above effect of GYY4137 (Figure S4c).

**FIGURE 6:**
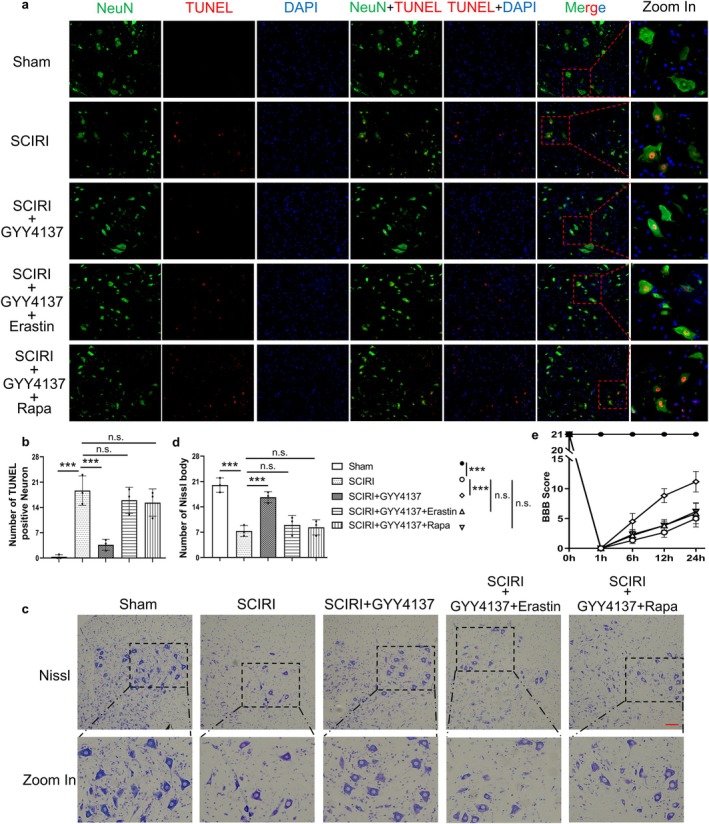
H_2_S alleviates neuronal cell death by inhibiting ferritinophagy‐mediated ferroptosis in SCIRI rats. (a) The NeuN and TUNEL immunofluorescence colocalization staining of the spinal cord in each group, red (TUNEL), green (NeuN) and blue (DAPI) (200×, scale bar = 100 μm). (b) Statistical analyses of the count of TUNEL‐positive neurons in the spinal cord (*n* = 3 rats per group). (c) The Nissl staining of the anterior horn of the spinal cord in each group (200×, scale bar = 100 μm). (d) Statistical analyses of Nissl bodies (*n* = 3 rats per group). (e) The BBB scores of different groups at each time point (*n* = 6 rats per group). ***p < 0.001; n.s., not significant.

Furthermore, we performed the TUNEL and NeuN immunofluorescence colocalization staining (Figure 6a). The results showed that TUNEL‐positive neurons were significantly reduced after GYY4137 treatment compared to the SCIRI rats, and there was no difference in GYY4137 combined with rapamycin or erastin compared to the SCIRI rats (Figure 6b). Meanwhile, we further tested Nissl staining and BBB scores (Figure 6c,e). As expected, GYY4137 significantly restored lower limb motor function and ameliorated the loss of neurons, but these effects disappeared when it was combined with rapamycin or erastin (Figure 6d,e). More generally, our results suggested that hydrogen sulfide attenuated neuronal death by inhibiting SCIRI‐induced ferritinophagy and ferroptosis.

## 4 Discussion

The results of the present study revealed that the SCIRI rats had significantly lower BBB scores and neuron numbers, and that these pathogeneses of SCIRI were involved with ferroptosis. Subsequent experiments showed that SCIRI‐induced ferroptosis with iron homeostasis disequilibrium, accumulation of labile iron pool (LIP) and peroxidation of lipids, all of which were dependent on NCOA4‐mediated ferritinophagy. Further experiments demonstrated that suppression of ferroptosis or ferritinophagy improved functional recovery. Notably, H_2_S significantly ameliorated neuron loss and restored neurological function in the SCIRI rats via inhibiting autophagic ferritin degradation, and ultimately inhibiting iron‐dependent ferroptosis. More generally, these findings suggested that ferritinophagy‐mediated ferroptosis might exert a novel role in the pathogenesis of SCIRI and that H_2_S might be an effective treatment for patients with SCIRI.

Mounting evidence supports the predominance of ferroptosis in the regulation of diseases [7, 37]. Moreover, the Slc7a11‐GSH‐GPx4 axis is the most studied anti‐ferroptosis mechanism at present [38]. In this study, we verified that the expression of GPx4 and Slc7a11 protein decreased, which indicated ferroptosis was activated after SCIRI. Immunohistochemical staining showed that GPx4 was predominantly expressed in the neurons, suggesting that ferroptosis was a cause of SCIRI. Meanwhile, Fer‐1 treatment (a typical ferroptosis inhibitor) could alleviate cell death and promote functional recovery. In this study, the findings suggested that SCIRI is tightly linked with ferroptosis, which at least partially is attributable to neuron death. SCIRI is clearly a dynamic process, which contains complicated mechanisms for cell death and is hard to attribute to one specific type of cell death. Thus, it requires more in‐depth research on the coordination and crossover of these cell death processes.

Interestingly, multiple pieces of evidence elucidated an important relationship between ferroptosis and autophagy [39, 40], which indicates its role in driving cells toward ferroptotic death [15, 16, 17]. It is regarded as an upstream mechanism that induces ferroptosis via the regulation of reactive oxygen species and cellular iron homeostasis [41]. Besides, we previously reported that SCIRI could induce autophagy, which contributes to neuron death [24]. In order to investigate the correlation and causation between ferroptosis and autophagy in SCIRI, we performed the experiments of loss of function and gain of function. The results demonstrated that autophagy interacted with ferroptosis in neuronal cells and determined that the ferritinophagy was required for neuron ferroptosis. Although ferritinophagy is involved in a variety of pathological processes, studies of expression of NCOA4 during ferritinophagy have not yielded uniform results [16, 42, 43]. According to the results of the present study, we inferred that the Fenton reaction occurred promptly in the early phase because of labile iron pool accumulation, triggered by direct iron influx and indirect degradation of Ferritin via autophagy facilitated by the large number of oxidants. With the continued increase of LIP in the cell, NCOA4 might be degraded gradually along with ferritin in the autolysosome through overwhelming autophagy. Importantly, we verified that H_2_S significantly reduced peroxidation of lipid and degradation of ferritin, and salvaged neuronal death. Collectively, these results suggested that overactivated NCOA4‐mediated ferritinophagy undermined iron homeostasis and boosted ferroptosis in neurons after SCIRI, and that H_2_S treatment could reverse these pathological processes.

Hydrogen sulfide has been recognized as a gasotransmitter molecule with a wide range of physiological function regulations, including, but not limited to, the cardiovascular, neuronal, and the function of ion channels [18, 44, 45, 46]. We previously reported that H_2_S could decrease the level of MDA and ROS in SCIRI rats [26]. Interestingly, these are closely involved with ferroptosis, so we deduced that H_2_S might be associated with ferroptosis. In this study, we observed that GYY4137, a sustained H_2_S‐releasing donor, remarkably rescued cell death and reversed ferroptosis by suppressing ferritinophagy. We also further activated autophagy or ferroptosis by a pharmacological inducer to validate the effects of H_2_S involved in ferroptosis and ferritinophagy.

Our findings and those of other researchers indicated that the cross‐talk between ferroptosis and ferritinophagy might be involved in SCIRI and supported that the ferritinophagy stabilization is crucial for ferroptosis regulation in neuronal cell death after SCIRI. Meanwhile, our results suggested that H_2_S plays multiple modulatory effects in the protection of neurons following SCIRI by regulating neuronal iron homeostasis disequilibrium, lipid peroxidation, and overactivation of ferroptosis and ferritinophagy.

## 5 Conclusions

In conclusion, the findings of our present study suggested that dysregulation of iron homeostasis mediated by ferritinophagy acted as a crucial factor in the etiopathogenesis of SCIRI. Labile iron pool and reactive oxygen species accumulation resulted in peroxidation of lipids, which in turn triggered ferroptosis in SCIRI, and this process had been proven to be dependent on NCOA4‐regulated ferritinophagy. Moreover, GYY4137 was found to ameliorate neuron loss and motor dysfunction by inhibiting neuron ferritinophagy‐mediated ferroptosis in rats with SCIRI. It provides important clues that this is a potential and feasible strategy to apply sustained release H_2_S donors as a clinical treatment for reversing neuron damage and improving the outcome of patients with SCIRI.

## Author Contributions

Lei Xie and Tengbo Yu conceived the study. Lei Xie and Xiao Xiao designed the research; Lei Xie, Hang Wu, and Qiuping He performed the experiments; Lei Xie and Weipeng Shi contributed essential reagents or tools. Lei Xie, Hang Wu, and Qiuping He analyzed the data. Lei Xie and Xiao Xiao wrote the paper. Tengbo Yu and Xiao Xiao provided suggestions. All authors have read and approved the article.

## Ethics Statement

All animal operations and care procedures were approved by the Qingdao University Laboratory Animal Welfare Ethics Committee (No. 20230820SD20231212042). All treatments were performed gently, all surgeries were performed under anesthesia, and all efforts were made to minimize animal suffering.

## Consent

All authors have reviewed the final version of the manuscript and approved it for publication.

## Conflicts of Interest

The authors declare no conflicts of interest.

## Supporting information


Appendix S1.

## Data Availability

The data used to support the findings of this study are available from the corresponding author upon reasonable request.
