# Supplementary material for: Hydrogen Sulfide Sustained Release Donor Alleviates Spinal Cord Ischemia–Reperfusion‐Induced Neuron Death by Inhibiting Ferritinophagy‐Mediated Ferroptosis
Source: CNS Neurosci Ther. 2025 Apr 1;31(3):e70366. doi: 10.1111/cns.70366 (PMC11960479; doi:10.1111/cns.70366)
Supplement: Supplementary file 1 — Appendix S1. [file CNS-31-e70366-s001.docx]

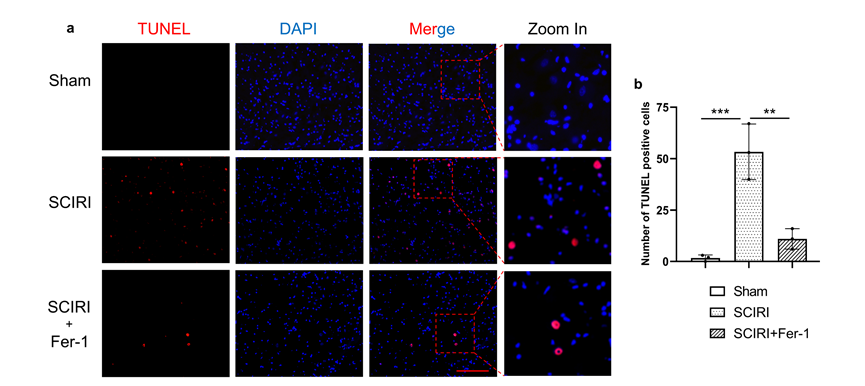


Figure S1 (a) Representative images of the spinal cord frozen sections labeled with TUNEL (red) and DAPI (blue) in each group (200×, scale bar = 100 μm). (b) Quantification of TUNEL‐positive cells counts in the ventral horn regions of the spinal cord. n = 3 rats per group. Data are presented as mean ± SD. *p < 0.05.


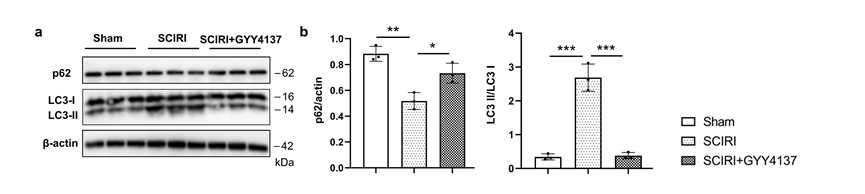


Figure S2 (a) Western blot showing expression level of autophagy-related proteins (p62 and LC3) of the spinal cord in each group. β-actin was used to ensure equal loading. (b) Densitometric analysis and quantification of p62/Actin and LC3Ⅱ/Ⅰ. n = 6 rats per group. Data are presented as mean ± SD. *p < 0.05; **p < 0.01.


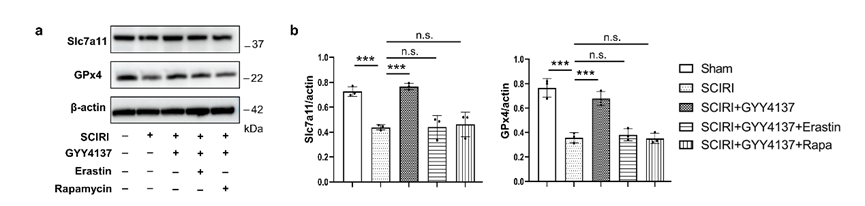


Figure S3 (a) Western blot showing expression level of ferroptosis-related proteins (Slc7a11 and GPx4) of the spinal cord in each group. β-actin was used to ensure equal loading. (b) Densitometric analysis and quantification of Slc7a11/Actin and GPx4/Actin (n = 6 rats per group). Data are presented as mean ± SD. *p < 0.05; **p < 0.01; ***p < 0.001; n.s., not significant.


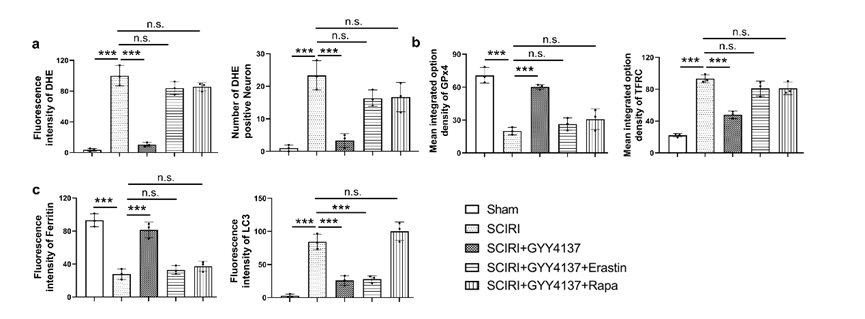


Figure S4 (a) Quantitative fluorescence intensity of DHE and DHE‐positive Neuron counts in the ventral horn regions of the spinal cord (n = 3 rats per group). (b) Analysis of the mean integrated option density of GPx4 and TFRC in each group (n = 3 rats per group). (c) Quantitative fluorescence intensity of Ferritin and LC3 in the ventral horn regions of the spinal cord (n = 3 rats per group). Data are presented as mean ± SD. *p < 0.05; **p < 0.01; ***p < 0.001; n.s., not significant.
